# Association of serum sclerostin and osteoprotegerin levels with the presence, severity and prognosis in patients with acute myocardial infarction

**DOI:** 10.1186/s12872-022-02654-1

**Published:** 2022-05-11

**Authors:** Xing Shui, Ruimin Dong, Zhen Wu, Zefeng Chen, Zheqi Wen, Leile Tang, Xujing Xie, Lin Chen

**Affiliations:** 1 Department of Cardiovascular Medicine, The Third Affiliated Hospital, SunYat-Sen University, No. 600, Tianhe Road, Guangzhou, 510630 China; 2 Department of Cardiac Care Unit, The Third Affiliated Hospital, SunYat-Sen University, Guangzhou, China

**Keywords:** Acute myocardial infarction, Sclerostin, Osteoprotegerin, Atherosclerosis, Prognosis

## Abstract

**Background:**

Bone-related proteins (such as sclerostin and osteoprotegerin [OPG]) are involved in the development of atherosclerosis. However, the relationship between bone-related proteins and acute myocardial infarction (AMI) has not been extensively evaluated. The purpose of this study was to assess the association of serum sclerostin and OPG with the presence, severity and prognosis in patients with AMI.

**Methods:**

This study prospectively enrolled 152 patients attacked by acute chest pain. Serum sclerostin and OPG were detected within the first 24 h after AMI diagnosis by ELISA kits. The AMI predictive efficacy of sclerostin and OPG were analyzed by receiver operating characteristics (ROC) curve. Univariable and multivariable linear regression analyses were performed to determine the association between bone-related proteins and scores indicating the severity of coronary artery occlusion. Moreover, prognostic values were assessed by Kaplan–Meier curves and Cox regression analysis.

**Results:**

There were 92 patients in AMI group, 60 in non-AMI group. Serum levels of sclerostin and OPG were significantly higher in AMI group than in non-AMI group (all *p* < 0.001), which showed predictive value for the presence of AMI (all *p* < 0.001). The area under the ROC curve values of sclerostin and OPG were 0.744 and 0.897, respectively. A multivariable linear regression analysis demonstrated that Ln-transformed sclerostin (β = 0.288, *p* = 0.009) and Ln-transformed OPG (Ln-OPG: β = 0.295, *p* = 0.019) levels were associated with GENISINI score, independently of conventional clinical parameters. In addition, Ln-OPG levels were still positively associated with GRACE score after adjustments (β = 0.320, *p* = 0.001). During a 1-year follow-up, patients above the median of sclerostin levels had higher incidence of major adverse cardiac events (MACE) than those below the median (*p* = 0.028). It was also observed that the MACE rates were higher in patients above the median of OPG levels, though no statistic importance (*p* = 0.060). After adjusting conventional risk factors by multivariate Cox regression, Ln-OPG was associated with incident MACE (hazard ratio = 2.188 [95% confidence intervals 1.102–4.344], *p* = 0.025).

**Conclusions:**

Bone-related proteins could exert a potential role in early risk stratification and prognosis assessment in patients with AMI.

## Background

Acute myocardial infarction (AMI) is a leading cause of death worldwide. Despite of the progress in early reperfusion and pharmacological agents, patients remain at high risk for morbidity and mortality after discharge [1]. Early and reliable identification and risk evaluation of AMI, which could prevent progression and improve outcomes, is still a major challenge. Although serial cardiac troponin tests have been considered as essential diagnostic steps to differentiate AMI, several limitations exist [2]. Some comorbidities like acute heart failure or chronic renal dysfunction are usually accompanied by elevated cardiac troponin in clinical practice [3, 4]. Meanwhile, the levels of cardiac troponin do not necessarily reflect the severity of coronary artery disease (CAD). Therefore, the identification of reliable biomarkers remains crucial for detecting the early stage and risk stratification of AMI.

Recently, a growing body of evidence show a close relationship between bone-related proteins and atherosclerosis, corresponding to crosstalk between bones and vessels, known as “bone-vessel axis” [5]. Osteoprotegerin (OPG), a member of the superfamily of tumor necrosis factor receptors, played its role in vascular injury, inflammation, and atherosclerosis [6]. Previous studies confirmed an association of the serum OPG levels with severity and prognosis of acute coronary syndrome (ACS) [7, 8]. Interestingly, another bone-related protein sclerostin, a negative regulator of the canonical wingless-type mouse mammary virus-integration site (Wnt) signaling pathways, has attracted attention. Since Wnt signaling pathways exerted a multifunctional role in endothelial dysfunction, the proliferation and migration of vascular smooth muscle cells (VSMCs), inflammation processes and vascular calcification progression, sclerostin was inevitably involved in the development of atherosclerosis [9]. Leto, et al. demonstrated sclerostin was presented in atherosclerotic plaques, mainly expressed by VSMCs compared to macrophages [10]. Nevertheless, reports focusing on the role played by sclerostin in atherosclerosis were inconclusive. Especially, few studies were available for elucidating the role of sclerostin in patients with AMI. As previous studies shed light on the relationship between serum OPG levels and AMI [7, 11], we aimed to explore the impact of serum sclerostin levels on AMI in present study, meanwhile, OPG was analyzed together as a potential positive control.

In action, a prospective study was conducted among patients admitted for acute chest pain. Serum levels of sclerostin and OPG were detected within the first 24 h after AMI diagnosis, and the predictive value of serum sclerostin and OPG for identification of AMI were further investigated. Meanwhile, correlation between these bone-related proteins and scores indicating the extent of coronary artery occlusion were evaluated. Finally, we analyzed the association between serum bone-related proteins levels and prognosis in patients with AMI.

## Methods

### Study population

A prospective cohort study was performed on patients hospitalized with acute chest pain in Chest Pain Center of our hospital from July 2018 to November 2019. All enrolled patients met the following inclusion criteria: first onset for admission; estimated glomerular filtration rate (eGFR) > 60 mL/min.1.73m^2^. Exclusion criteria: age < 18 or ≥ 80 years, thyroid disease, autoimmune disease, bone disease, severe liver dysfunction and malignant tumors, or current use of drug that could influence bone metabolism like hormones, bisphosphonates, and calcium supplements. Acute chest pain provoked by trauma, drugs or medical intervention was also excluded. This study was in line with the medical ethics standards of the 1975 Declaration of Helsinki and approved by the Ethics Committee of The Third Affiliated Hospital, Sun Yat-sen University. Written informed consent was obtained from each patient.

### Adjudicated final diagnosis

18-leads electrocardiography (ECG) was recorded from each patient to detect suspected myocardial infarction, meanwhile serial serum levels of cardiac troponin I (cTNI) (AQT90 FLEX, Copenhagen, Demark) were measured, and the normal range was 0.010–0.023 ng/mL following the manufacturer’s instructions. Diagnostic criteria of AMI were in accordance with the fourth universal definition of myocardial infarction (2018). In brief, AMI was diagnosed when there were symptoms of myocardial ischemia, or serial ischemic changes of ECG, and with detection of a rise and/or fall of cTN values with at least one above of the 99^th^ percentile URL [12]. The final diagnosis was judged by two independent cardiologists. Once AMI was established, an emergent or early percutaneous coronary intervention (PCI) indication was decided and performed by two experienced interventional cardiologists. According to the final diagnosis, patients hospitalized with acute chest pain were divided into AMI-group and non-AMI group.

### Clinical data collection and biochemical analysis

Demographic and clinical data, including age, sex, height, weight, systolic and diastolic blood pressure, heart rate and comorbidities, were collected at admission. Body mass index (BMI) was calculated from weight in kg divided by height in m^2^. Hypertension was diagnosed according to ESC/European Society of Hypertension guidelines [13]. Diabetes was defined based on the criteria of current guidelines [14]. Smoking status was defined as a person who smoked at admission or who had quit smoking within the year before admission.

Fasting blood samples were obtained within the first 24 h after AMI diagnosis and were centrifuged within 20 min of collection at 4 °C.Serum sclerostin and OPG levels were quantified using commercially available sandwich-type enzyme-linked immunoassay (ELISA) kits (RayBiotech Inc., Georgia, USA) following the manufacturer’s instructions. Meanwhile, analyses of biochemical indicators, including routine blood analysis (Sysmex XN-2000, Kobe, Japan), analysis of liver and renal function, and quantification of the levels of blood lipids profiles, blood glucose and uric acid (UA) (Hitachi 7600, Tokyo, Japan) were performed in laboratory medicine as soon as possible. Non-HDL-C levels were calculated from total cholesterol (TC) minus high density lipoprotein cholesterol (HDL-C) levels. Serial measurement of serum cTNI and N terminal pro B type natriuretic peptide (NT-proBNP) (AQT90 FLEX, Copenhagen, Demark) were performed to detect peak values within 3 days in AMI group. In brief, serum levels of cTNI and NT-proBNP were analyzed from samples collected before and after PCI, at 24 h, 48 h and 72 h after AMI diagnosis.

### Coronary angiography and severity of coronary artery occlusion

Selective coronary angiography was performed in patients with AMI using the standard Judkin’s technique. The severity of coronary artery lesion was determined by the GENSINI score system. According to the degree of luminal narrowing, severity scores reflecting angiographic stenosis of the coronary artery segment were 1, 2, 4, 8, 16, and 32 for 0–25%, 26–50%, 51–75%, 76–90%, 91–99%, and 100%, respectively [15]. The Global Registry of Acute Coronary Event (GRACE) was widely used to risk stratification for ACS in acute phase [16]. For each AMI patient, GRACE score was calculated using online calculator on admission or within 24 h of admission (http://www.outcomes-umassmed.org/grace) by following variables: age, systolic blood pressure, heart rate, creatinine value, Killip class, cardiac arrest at admission, ST-segment deviation in ECG, and abnormal cardiac markers.

### Follow-up

All patients with AMI were treated with standard secondary prevention medication as guideline-recommended, unless contraindications. Follow-up was prospectively conducted by telephone call or clinic visit of our hospital every 3 months after discharge. The occurrence of major adverse cardiovascular events (MACE) and the time were recorded. MACE was considered as non-fatal ischemic or hemorrhagic stroke, non-fatal myocardial infarction, hospitalized unstable angina pectoris, unplanned revascularization including PCI and coronary artery bypass grafting (CABG), and cardiac death. Patients who died of non-cardiovascular diseases were divided into loss to follow-up.

### Statistical analysis

Statistical analyses were performed using the SPSS 25.0 software (IBM Corporation, Armonk, NY, USA). Continuous variables were expressed as mean ± standard deviation (SD), or median (interquartile range), as appropriate. Categorical variables were expressed as the number (percentage). Differences of continuous variables were assessed by the student’s t-test or Mann–Whitney U-test, as appropriate. Differences of categorical variables were evaluated by Chi-square test. Receiver operating characteristics (ROC) curve and area under the ROC curve (AUC) were used to identify patients with AMI by MedCalc 20.0.3 software (MedCalc, Ostend, Belgium). Since the distribution of serum sclerostin, OPG, fasting plasma glucose (FPG) and triglyceride (TG) levels showed positive skewing, they were logarithmically (Ln) transformed and were applied in correlation and regression analysis. The Spearman’s correlation was used to assess the correlation of bone-related proteins (serum sclerostin and OPG) with cTNI levels on admission in patients enrolled. The Pearson’s correlation was used to study the relationships between logarithmically transformed bone-related proteins and scores indicating the severity of coronary artery occlusion in patients with AMI (GENSINI score and GRACE score). The univariate linear regression was performed to evaluate the non-adjusted relationships of clinical parameters and severity scores. Significant variables in univariate linear regression (*p* < 0.15) were included in the multivariable linear regression model, and only variables with significance were shown in table. The event rate of MACE during a 1-year follow-up was plotted in Kaplan–Meier curves based on serum sclerostin and OPG concentrations (below and above the median), and differences in survival curves were evaluated by the log-rank test. The independent association between bone-related proteins and MACE was investigated by univariate and multivariate Cox regression analyses. Variables that were significantly associated with MACE in univariate Cox regression (*p* < 0.15) were included in multivariate Cox regression analysis with only variables with significance were shown in table. The C-index was calculated to compare the prognostic value of cTNI on admission with bone-related proteins in univariate Cox regression analysis by R Studio, version 3.5.1. A two-tailed *p* value < 0.05 was considered statistically significant.

## Results

### Baseline characteristics of the study subjects

As shown in Table 1, there were 92 patients in AMI group, 60 in non-AMI group, including 28 cases of unstable angina, 25 cases of stable angina, 3 cases of myocarditis, 2 cases of hypertrophic cardiomyopathy and 2 cases of myocardial bridge. In patients with AMI, the serum levels of cTNI on admission, sclerostin and OPG (all *p* < 0.001) were significantly higher than those in non-AMI group. More males (*p* = 0.012) and smokers (*p* = 0.045) were seen in AMI group when compared with non-AMI group. Lower systolic and diastolic blood pressure (all *p* < 0.001) at admission were observed in AMI group, which may due to impaired heart function. Meanwhile, TC, low density lipoprotein cholesterol (LDL-C), Non-HDL-C, lipoprotein(a), FPG and white blood cell (WBC) count were significantly higher in patients with AMI, as compared with non-AMI patients. Furthermore, lower HDL-C levels were noticed in AMI group. However, there were no significant differences in age, BMI, history of hypertension and diabetes, heart rate, TG, hemoglobin A1c (HbA1c), creatinine, eGFR and UA between two groups.

**Table 1:** Baseline demographic and clinical characteristics of subjects

Variables	AMI (n = 92)	Non-AMI (n = 60)	*p* value
Age, years	56.8 ± 10.7	54.2 ± 10.2	0.140
Male, n (%)	75 (81.5)	38 (63.3)	0.012*
BMI, kg/m^2^	25.1 ± 2.9	25.5 ± 3.8	0.456
Hypertension, n (%)	47 (51.1)	34 (56.7)	0.500
Diabetes, n (%)	24 (26.1)	21 (35.0)	0.239
Smoking status, n (%)	61 (66.3)	30 (50.0)	0.045*
SBP, mmHg	130.1 ± 22.6	143.2 ± 20.3	< 0.001*
DBP, mmHg	80.3 ± 14.1	91.6 ± 15.4	< 0.001*
Heart rate, bpm	81.3 ± 15.0	81.1 ± 10.8	0.939
**Laboratory tests**			
cTNI on admission, ng/mL	0.17 (0.01, 1.28)	0.01 (0.01, 0.01)	< 0.001*
Total cholesterol, mmol/L	4.93 ± 1.29	4.52 ± 1.11	0.048*
Triglyceride, mmol/L	1.63 (1.13, 2.46)	1.82 (0.99, 2.71)	0.865
HDL-C, mmol/L	0.91 ± 0.21	0.99 ± 0.22	0.017*
LDL-C, mmol/L	3.33 ± 1.08	2.93 ± 1.00	0.022*
Non-HDL-C, mmol/L	4.03 ± 1.24	3.53 ± 1.13	0.014*
Lipoprotein(a), mg/L	195.50 (101.50, 356.75)	124.50 (64.00, 266.00)	0.009*
FPG, mmol/L	6.15 (5.37, 8.06)	5.22 (4.65, 5.76)	< 0.001*
HbA1c, %	6.50 ± 1.70	6.03 ± 1.28	0.074
Creatinine, umol/L	73.98 ± 17.03	75.50 ± 19.15	0.609
eGFR, mL/min/1.73m^2^	93.07 ± 17.16	96.21 ± 17.83	0.282
Uric acid, umol/L	409.04 ± 117.38	428.24 ± 106.25	0.317
WBC count, × 10E9	11.18 ± 3.72	6.91 ± 1.55	< 0.001*
OPG, pg/mL	105.98 (79.79, 166.05)	56.79 (48.77, 69.66)	< 0.001*
Sclerostin, pg/mL	526.31 (355.19, 776.84)	325.39 (237.10, 455.76)	< 0.001*

*AMI* acute myocardial infarction, *BMI* body mass index, *SBP* systolic blood pressure, *DBP* diastolic blood pressure, *cTNI* cardiac troponin I, *HDL-C* high density lipoprotein cholesterol, *LDL-C* low density lipoprotein cholesterol, *FPG* fasting plasma glucose, *HbA1c* hemoglobin A1c, *eGFR* estimated glomerular filtration rate, *WBC* white blood cell, *OPG* osteoprotegerin

*p < 0.05 was considered statistically significant

### Predictive value of serum sclerostin and OPG for identifying AMI in patients with acute chest pain

**Fig. 1:**
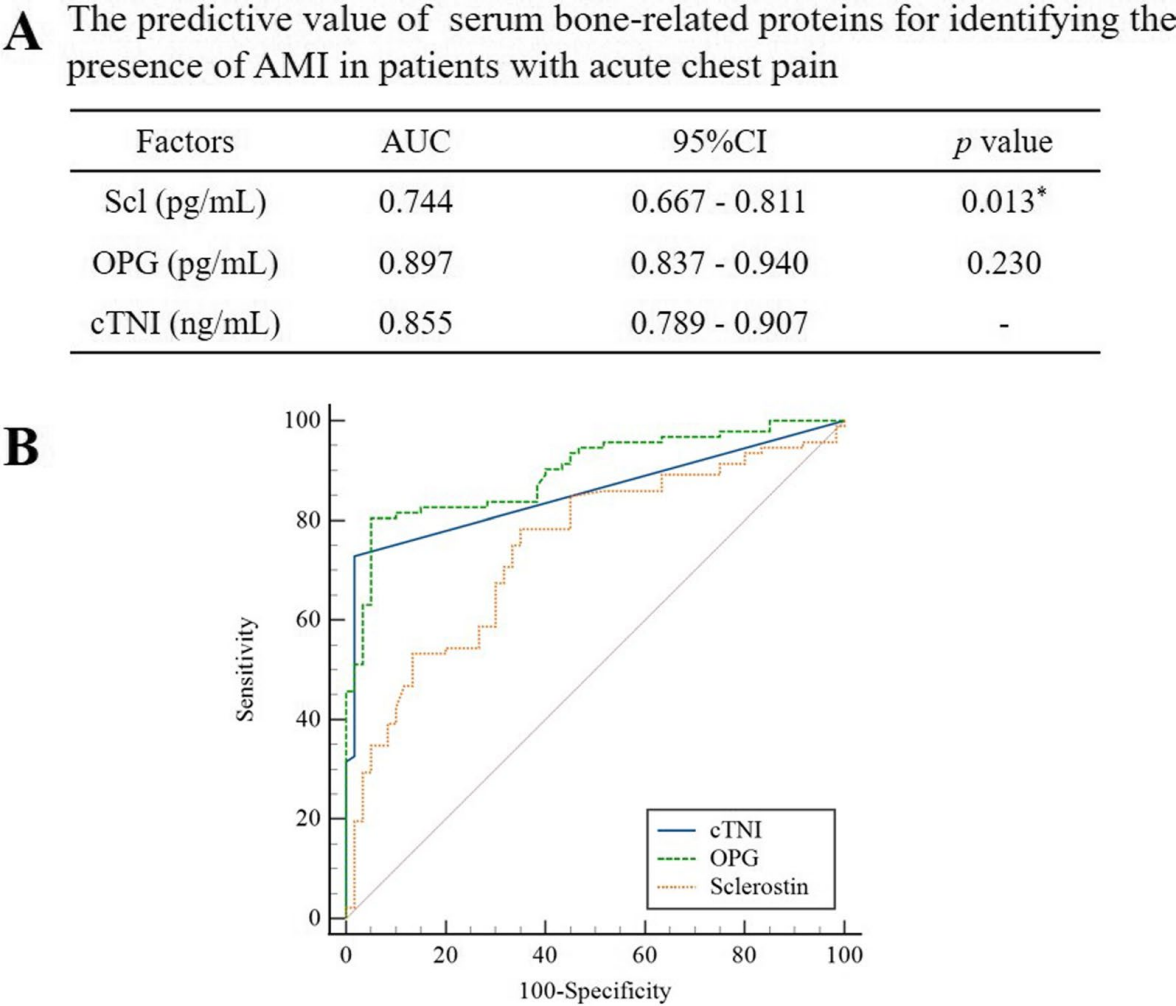
The predictive ability of serum bone-related proteins for AMI among patients with acute chest pain. **a** Predictive value. AMI: acute myocardial infarction, Scl: sclerostin, OPG: osteoprotegerin, cTNI: cardiac troponin I, AUC: area under the curve, CI: confidence intervals. **b** ROC curve. ROC: receiver operating characteristics. *p < 0.05 was considered statistically significant when compared with cTNI on admission

Both of serum levels of Ln-sclerostin (r = 0.347) and Ln-OPG (r = 0.518) were positively correlated with cTNI levels on admission (all *p* < 0.001). ROC curve was conducted to compare the efficacy of serum sclerostin and OPG for differentiating AMI in patients with acute chest pain (shown in Fig. 1). Both of serum bone-related proteins (sclerostin and OPG) and cTNI on admission had predictive value for identifying the presence of AMI (all *p* < 0.001). The AUC values of serum sclerostin, OPG and cTNI on admission were 0.744, 0.897, and 0.855, respectively. The efficiency of serum OPG levels for identification of AMI was comparable to that of cTNI on admission (*p* = 0.230). However, it was found that AUC_sclerostin_ was lower than AUC_cTNI_ (*p* = 0.013).

### Associations between serum bone-related proteins and scores indicating the extent of coronary artery occlusion

**Fig. 2:**
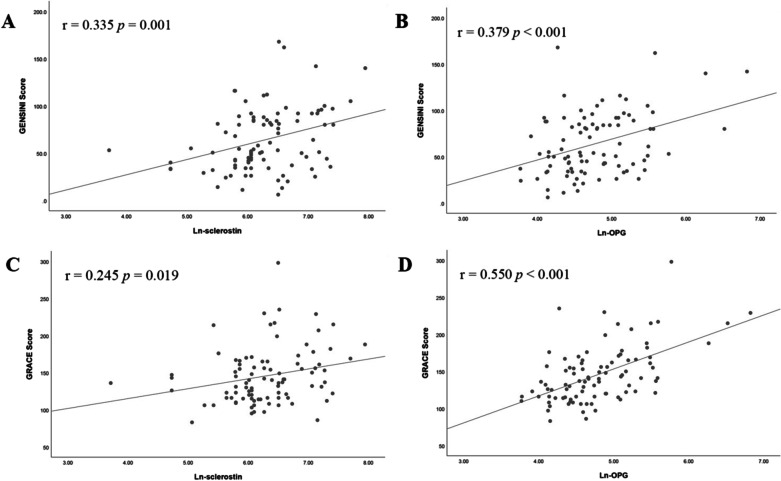
Correlation between serum bone-related proteins and scores indicating the severity of coronary artery occlusion. **a** Ln-sclerostin and GENSINI score **b** Ln-OPG and GENSINI score **c** Ln-sclerostin and GRACE score **d** Ln-OPG and GRACE score. Data of sclerostin and OPG showed skewed distribution and therefore were Ln-transformed before Pearson’s correlation analysis. GRACE: the global registry of acute coronary event, OPG: osteoprotegerin. *p* < 0.05 was considered statistically significant

Univariate linear regression analysis indicated that BMI (*p* = 0.039), Ln-sclerostin (*p* = 0.001), and Ln-OPG (*p* < 0.001) levels were significantly related to values of GENSINI score. In multivariable linear regression models, Ln-sclerostin (*p* = 0.009) and Ln-OPG (*p* = 0.019) levels remained positively associated with GENSINI score, respectively (shown in Table 2, Table 3, and Fig. 2). The GRACE score, which was widely used as a risk prediction tool in patients with ACS, was positively correlated with diabetes (*p* = 0.001), age (*p* < 0.001), Ln-FPG (*p* = 0.007), Ln-sclerostin (*p* = 0.019), and Ln-OPG (*p* < 0.001), and negatively correlated with BMI (*p* = 0.018), LDL-C (*p* = 0.003), Ln-TG (*p* < 0.001) and eGFR (*p* < 0.001) in univariate linear regression analysis. After adjusting adopted variables, Ln-OPG levels were still positively associated with GRACE score (*p* = 0.001).

**Table 2:** Linear regression analyses evaluating the relationships between Ln-sclerostin and scores indicating the severity of coronary artery occlusion

Variables	GENSINI score				GRACE score			
	Univariate		Multivariate		Univariate		Multivariate	
	β (SE)	*p* value	β (SE)	*p* value	β (SE)	*p* value	β (SE)	*p* value
Male	−0.009 (0.104)	0.931	–	–	−0.193 (0.103)	0.065	–	–
Hypertension	−0.048 (0.106)	0.651	–	–	0.021 (0.105)	0.842	–	–
Diabetes	0.116 (0.106)	0.275	–	–	0.349 (0.099)	0.001*	–	–
Smoking status	−0.142 (0.105)	0.181	–	–	−0.174 (0.104)	0.097	–	–
Age, years	0.041 (0.106)	0.699	–		0.588 (0.085)	< 0.001*	0.358 (0.106)	0.001*
BMI, kg/m^2^	−0.216 (0.103)	0.039*	−0.252 (0.107)	0.021*	−0.246 (0.102)	0.018*	–	–
Ln-FPG, mmol/L	0.165 (0.105)	0.118	–	–	0.280 (0.101)	0.007*	–	–
HDL-C, mmol/L	−0.031 (0.107)	0.773	–	–	−0.124 (0.105)	0.240	–	–
LDL-C, mmol/L	−0.023 (0.104)	0.825	–	–	−0.310 (0.100)	0.003*	–	–
Ln-TG, mmol/L	−0.009 (0.112)	0.936	–		−0.386 (0.097)	< 0.001*	−0.201 (0.092)	0.032*
UA, umol/L	0.171 (0.108)	0.120	–	–	0.027 (0.108)	0.804	–	–
eGFR, mL/min/1.73m^2^	−0.093 (0.106)	0.381	–	–	−0.485 (0.092)	< 0.001*	–	–
WBC count, × 10E9	0.052 (0.106)	0.626	–	–	0.025 (0.106)	0.814	–	–
Ln-sclerostin, pg/mL	0.335 (0.100)	0.001*	0.288 (0.108)	0.009*	0.245 (0.102)	0.019*	–	–

Data of sclerostin, triglyceride, and fasting plasma glucose showed skewed distribution and therefore were Ln-transformed before analysis

Significant variables (*p* < 0.15) in univariate linear regression were further included in the multivariable linear regression, and only variables with significance were shown in table. Adopted variables: BMI, Ln-FPG, UA, and Ln-sclerostin for multivariate regression model of GENSINI score; male, diabetes, smoking status, age, BMI, Ln-FPG, LDL-C, Ln-TG, eGFR and Ln-sclerostin for multivariate regression model of GRACE score

*BMI* body mass index, *FPG* fasting plasma glucose, *HDL-C* high density lipoprotein cholesterol, *LDL-C* low density lipoprotein cholesterol, *TG* triglyceride, *UA* uric acid, *eGFR* estimated glomerular filtration rate, *WBC* white blood cell, *GRACE* the global registry of acute coronary event

*p < 0.05 was considered statistically significant

**Table 3:** Linear regression analyses assessing the relationships between Ln-OPG and scores indicating the severity of coronary artery occlusion

Variables	GENSINI score				GRACE score			
	Univariate		Multivariate		Univariate		Multivariate	
	β (SE)	*p* value	β (SE)	*p* value	β (SE)	*p* value	β (SE)	*p* value
Male	−0.009 (0.104)	0.931	–	–	−0.193 (0.103)	0.065	–	–
Hypertension	−0.048 (0.106)	0.651	–	–	0.021 (0.105)	0.842	–	–
Diabetes	0.116 (0.106)	0.275	–	–	0.349 (0.099)	0.001*	–	–
Smoking status	−0.142 (0.105)	0.181	–	–	−0.174 (0.104)	0.097	–	–
Age, years	0.041 (0.106)	0.699	–		0.588 (0.085)	< 0.001*	0.362 (0.100)	0.001*
BMI, kg/m^2^	−0.216 (0.103)	0.039*			−0.246 (0.102)	0.018*	–	–
Ln-FPG, mmol/L	0.165 (0.105)	0.118	–	–	0.280 (0.101)	0.007*	–	–
HDL-C, mmol/L	−0.031 (0.107)	0.773	–	–	−0.124 (0.105)	0.240	–	–
LDL-C, mmol/L	−0.023 (0.104)	0.825	–	–	−0.310 (0.100)	0.003*	–	–
Ln-TG, mmol/L	−0.009 (0.112)	0.936	–		−0.386 (0.097)	< 0.001*	−0.174 (0.087)	0.049*
UA, umol/L	0.171 (0.108)	0.120	–	–	0.027 (0.108)	0.804	–	–
eGFR, ml/min/1.73m^2^	−0.093 (0.106)	0.381	–	–	−0.485 (0.092)	< 0.001*	–	–
WBC count, × 10E9	0.052 (0.106)	0.626	–	–	0.025 (0.106)	0.814	–	–
Ln-OPG, pg/mL	0.379 (0.098)	< 0.001*	0.295 (0.124)	0.019*	0.550 (0.088)	< 0.001*	0.320 (0.095)	0.001*

Data of osteoprotegerin, triglyceride, and fasting plasma glucose showed skewed distribution and therefore were Ln-transformed before analysis

Significant variables (*p* < 0.15) in univariate linear regression were further included in the multivariable linear regression, and only variables with significance were shown in table. Adopted variables: BMI, Ln-FPG, UA, and Ln-OPG for multivariate regression model of GENSINI score; male, diabetes, smoking status, age, BMI, Ln-FPG, LDL-C, Ln-TG, eGFR and Ln-OPG for multivariate regression model of GRACE score

*BMI* body mass index, *FPG* fasting plasma glucose, *HDL-C* high density lipoprotein cholesterol, *LDL-C* low density lipoprotein cholesterol, *TG* triglyceride, *UA* uric acid, *eGFR* estimated glomerular filtration rate, *WBC* white blood cell, *OPG* osteoprotegerin, *GRACE* the global registry of acute coronary event

*p < 0.05 was considered statistically significant

### Cox regression analysis for relationship between bone-related proteins and MACE

**Fig. 3:**
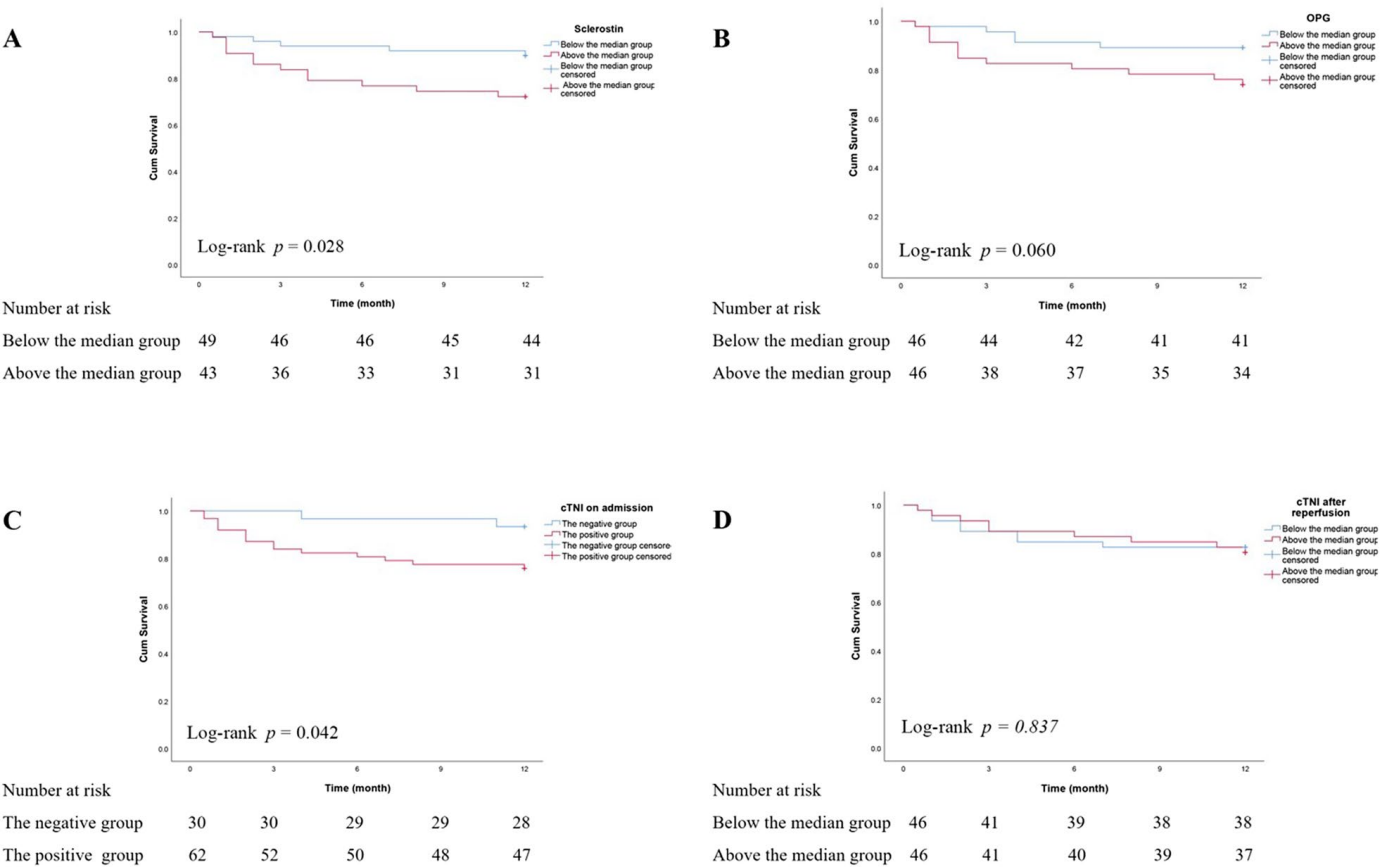
Kaplan–Meier estimates of cumulative survival of AMI patients according to serum sclerostin levels (**a**), OPG levels (**b**), cTNI levels on admission (**c**) and peak values of cTNI after reperfusion (**d**). The continuous cTNI values on admission were transformed into the positive and the negative group based on the threshold values of 0.023 ng/mL. Bone-related proteins and the peak values of cTNI after reperfusion were divided into two groups: below and above the median group. (median sclerostin:526.31 pg/mL, median OPG:105.98 pg/mL, and median cTNI after reperfusion:25.07 ng/mL). AMI: acute myocardial infarction, OPG: osteoprotegerin, cTNI cardiac troponin I. *p* < 0.05 was considered statistically significant

During the follow-up period of 12 months, a total of 17 events occurred in patients with AMI: cardiac death (n = 3), non-fatal MI during follow up (n = 3), non-fatal stroke (n = 1), unplanned revascularization (PCI/CABG) (n = 5) and hospitalized unstable angina pectoris (n = 5). The median values of serum sclerostin and OPG levels at baseline were 526.31 pg/mL and 105.98 pg/mL. Patients with AMI were stratified into two groups according to the bone-related proteins above and below the median, respectively. The Kaplan–Meier analysis was used to assess the cumulative survival rate. As compared with patients below the median, the MACE rates were clearly higher in patients above the median of sclerostin levels (*p* = 0.028). It was also observed that patients above the median of OPG levels had higher MACE rates than those below the median, though no statistic importance (*p* = 0.060). According to the threshold values of cTNI on admission, patients with AMI were assigned into the cTNI-positive group (cTNI > 0.023 ng/mL) and the cTNI-negative group (cTNI ≤ 0.023 ng/mL). It was found that patients in the cTNI-positive group had higher MACE rates than that of the cTNI-negative group (*p* = 0.042). Nevertheless, the peak values of cTNI after reperfusion were not significantly associated with the MACE rates (*p* = 0.837) (shown in Fig. 3). In univariate Cox regression analysis, Ln-OPG was a significant predictor of incident MACE (hazard ratio 2.932 [95% confidence intervals 1.532–5.610], *p* = 0.001). However, Ln-sclerostin was not significantly associated with MACE (hazard ratio 1.887 [95% confidence intervals 0.890–4.002], *p* = 0.098). The C-index of cTNI on admission, Ln-sclerostin and Ln-OPG for predicting prognosis were 0.623, 0.636, and 0.703, respectively. After adjusting adopted factors by multivariate Cox regression, Ln-OPG remained associated with incident MACE (hazard ratio 2.188 [95% confidence intervals 1.102–4.344], *p* = 0.025) (shown in Table 4).

**Table 4:** Cox regression analysis of serum OPG levels for MACE in patients with AMI

Variables	Univariate analysis HR (95% CI)	*p* value	Multivariate analysis HR (95% CI)	*p* value
Age, years	1.020 (0.973–1.070)	0.406	–	–
Gender	0.692 (0.226–2.124)	0.520	–	–
Comorbidities	2.235 (0.728–6.857)	0.160	–	–
Smoking status	0.676 (0.257–1.778)	0.428	–	–
BMI, kg/m^2^	0.850 (0.721–1.001)	0.052		
cTNI on admission, ng/mL	4.074 (0.931–17.821)	0.062		
NT-proBNP, ng/mL	2.727 (0.784–9.492)	0.115		
Ln-OPG, pg/mL	2.932 (1.532–5.610)	0.001*	2.188 (1.102–4.344)	0.025^*^

Comorbidities indicated hypertension, diabetes

Data of osteoprotegerin showed skewed distribution and therefore was Ln-transformed before analysis

The continuous cTNI levels on admission were transformed into the positive and the negative values based on the threshold value of 0.023 ng/mL. Peak values of NT-proBNP were categorized into the positive and negative values according to the threshold value of 300 pg/mL in acute phase

Significant variables (*p* < 0.15) in univariate Cox regression were further included in the multivariable Cox regression, and only variables with significance were shown in table. Adopted factors: BMI, cTNI on admission, peak values of NT-proBNP, and Ln-OPG

*OPG* osteoprotegerin, *MACE* major adverse cardiac events, *AMI* acute myocardial infarction, *BMI* body mass index, *cTNI* cardiac troponin I, *NT-proBNP* N terminal pro B type natriuretic peptide, *HR* hazard ratio, *CI* confidence intervals

*p < 0.05 was considered statistically significant

## Discussion

In the present study, our results demonstrated that compared with non-AMI subjects, serum levels of sclerostin and OPG were higher in patients with AMI. Both showed predictive values for identification of AMI among patients with acute chest pain. Meanwhile, it was observed that serum sclerostin and OPG levels were associated with GENSINI score, which could reflect the severity of coronary artery occlusion, independently of conventional clinical parameters. In addition, serum OPG levels remained associated with GRACE score after adjustments, which could reflect the risk of mortality in hospitalization of patients with ACS. Moreover, patients above the median of bone-related proteins were accompanied with high incidence of MACE during 1 year follow-up. Furthermore, serum OPG levels remained associated with incident MACE after adjusting conventional risk factors.

Sclerostin, a soluble glycoprotein, was potent inhibitor of Wnt signaling by binding to the co-receptor composed of low-density lipoprotein receptor-related proteins 5 (LRP5) or LRP6 and the Frizzled family proteins [17]. Since accumulating evidence showed the important roles of the Wnt signaling pathways in pathogenesis of atherosclerosis, serum sclerostin was proposed to exert an endocrine role in atherosclerosis [18]. Although circulating sclerostin was expressed mainly by osteocytes [19], Leto et al. demonstrated the expression of sclerostin in VSMCs of atherosclerotic plaques from patients who underwent carotid endarterectomy [10]. Afterwards, Annelies et al. also provided evidence for the local vascular action of sclerostin both in human and rat calcified aortas, meanwhile sclerostin levels were increased in calcifying VSMCs [20]. As VSMCs were considered as major precursors contributing to osteochondrogenesis and calcification in atherosclerosis [21], it was speculated that serum sclerostin may play an endocrine modulatory role in atherosclerosis development. Indeed, a case control study was conducted to detect the levels of sclerostin in Egyptian female patients with type 2 diabetes with and without atherosclerosis, they found a positive relationship between sclerostin and atherosclerosis in patients with type 2 diabetes [22]. Furthermore, Shalash et al. suggested that elevated serum sclerostin levels were independently associated with subclinical atherosclerosis in subjects with type 2 diabetes [23]. Interestingly, Ghardashi-Afousi et al. indicated 12 weeks of high-intensity interval training decreased serum sclerostin levels in patients with type 2 diabetes, meanwhile improved carotid intima-media thickness, a surrogate marker of atherosclerosis, was observed [24]. However, Krishna et al. assessed the possible role of sclerostin on atherosclerosis progression in AngII-infused ApoE^−/−^ mice, they found elevated sclerostin could suppress intimal plaque formation in the aortic arch, exerting a protective role on atherosclerosis progression [25]. Further studies are required to push forward the role of sclerostin in the pathogenesis of atherosclerosis. Until now, few studies were available for elucidating the impact of serum sclerostin on AMI. In present study, we demonstrated that serum sclerostin levels were significantly higher in patients with AMI, indicating a statistically predictive value for identifying AMI among patients with acute chest pain. However, the predictive efficacy of sclerostin seemed to be inferior to cTNI on admission. Meanwhile, significantly positive association between sclerostin and GENSINI score was observed, which could reflect the severity of coronary artery occlusion. Moreover, patients above the median of serum sclerostin levels presented poor prognosis in 1 year follow-up after discharge. Additionally, we found patients in the cTNI-positive group on admission were associated with high MACE rates, nevertheless, the peak values of cTNI after PCI were not associated with the high incidence of MACE. This was in consistence with what has been found in previous study by Jose, et al. They observed that high-sensitivity cTNT on admission, rather than post-reperfusion, was independently associated with prognosis after an average 53 months follow-up period in patients with AMI undergoing primary PCI [26]. The C-index was performed to preliminarily compare the prognostic value of sclerostin and cTNI on admission, the efficacy of sclerostin appeared to be comparable to cTNI on admission, whereas, further studies are needed to validate this finding. Although the multivariate analysis did not obtain a relationship between serum sclerostin and GRACE score, we assume that serum sclerostin could be used to complement risk stratification for AMI.

Nevertheless, He et al. investigated the relationship between serum sclerostin levels and adverse outcomes in elderly patients with stable CAD who were undergoing PCI [27]. After a 3-year follow-up, they observed the higher serum sclerostin levels were associated with better outcomes after PCI. Age ≥ 65 years and eGFR ≥ 45 mL/min.1.73m^2^ met the inclusion criteria. The SYNTAX score was used to assess severity of CAD, however, the direct correlation analysis between sclerostin levels and SYNTAX score was not available. Another observational study was also performed to assess the relation of serum sclerostin with atherosclerosis severity by SYNTAX score in patients with stable coronary artery disease or ACS, but the results failed to support direct relationship between sclerostin and CAD severity [28]. Recently, Kern et al. investigated the impact of baseline sclerostin levels on 9-years outcomes in patients without significant renal function impairment and undergoing coronary angiography [29]. 205 patients with a mean age of 62.9 ± 0.6 years were enrolled. They showed that the high sclerostin (above median) group presented statistically significant higher rates of MACE and death, implying subjects with above median sclerostin levels might have a worse prognosis. Afterwards, in subgroup analysis, the chronic coronary syndrome subgroup, rather than the ACS subgroup showed statistically significant impact of sclerostin levels on MACE and death. Possible explanations for the discrepancy were the feature of subjects enrolled, evaluation tools, baseline kidney function, and follow-up times. In our study, follow-up subjects comprised of ST-elevated (64.1%) and non-ST-elevated (35.9%) myocardial infarction undergoing PCI with average age 56.8 ± 10.7 years and eGFR ≥ 60 mL/min.1.73 m^2^. The severity of CAD was evaluated by traditional GENSINI score rather than SYNTAX score. Notably, AMI may occur when an atherosclerotic plaque erosions or ruptures, which was different from stable CAD condition [12]. We assumed that the more severe the atherosclerotic plaque ruptured, the more sclerostin was released from the calcified VSMCs, which might be related to the extent of vulnerability of plaque in acute phase. Moreover, due to the heterogeneity in enrolled subject cohorts, the impact of serum sclerostin on clinical outcome in chronic kidney disease remains controversial. However, Zou et al. found that low serum sclerostin was related with better overall survival in patients with peritoneal dialysis, which was consistent with ours [30]. Furthermore, only first 1-year follow-up after discharge was observed in present study, which itself was the high prevalence period of MACE. As the real contribution of sclerostin to the development of atherosclerosis is still uncertain, further mechanistic and clinical studies are needed to confirm our speculation.

Increasing evidence indicated that OPG levels were associated with CAD [6, 31]. In present study, we confirmed that serum OPG levels had predictive value of AMI, which was comparable to cTNI on admission. Meanwhile, serum OPG levels were positively associated with GENSINI score and GRACE score, indicating the predictive value of severity of AMI, which was consistent with previous studies [8]. Moreover, Fuernau et al. suggested serum OPG levels collected 24 h after infarction were independent predictors of MACE in acute STEMI patients [11]. In our study, we also validated that serum OPG was associated with incident MACE in patients with AMI after discharge by adjusting conventional risk factors. The C-index was applied to preliminarily compare the prognostic value of serum OPG and cTNI on admission, the efficacy of OPG seemed to be superior to cTNI on admission. Conversely, insights from the PLATO (Platelet Inhibition and Patient Outcomes) trial showed plasma OPG was not an independent marker of ischemic cardiovascular events in patients with ACS [32]. Explanation for the discrepancy may be different reagent test kit. Subsequent studies warranted to understand the complex interrelations between OPG and atherosclerosis.

Nevertheless, there are several limitations in present study. First, we are not able to obtain a direct cause-and-effect relationship between bone-related proteins and clinical events due to the observation study design. Second, residual confounders are hard to avoid. For example, diet, excise, medication compliance, and osteoporosis were not monitored during the study. Third, since serum sclerostin and OPG were measured only once within first 24 h after MI diagnosis, changes in these proteins in response of treatment and disease progression could not be assessed. Fourth, we noticed the impact of age, postmenopausal women, and history of diabetes on the serum levels of sclerostin [18, 19]. In present study, there were no significant differences in age and history of diabetes between AMI and non-AMI groups. Women commonly suffered from AMI after postmenopausal period due to possibly estrogen protection. In our study, all of them were postmenopausal women. Fifth, the study population was relatively small, further large prospective studies with well-characterized population are required to confirm the role played by serum sclerostin and OPG in the development and progression of AMI.

## Conclusions

Our study suggests that the assessment of serum sclerostin and OPG could be useful for the identification of AMI in patients with acute chest pain. Meanwhile, both could reflect the severity of coronary artery occlusion. In addition, serum OPG could assist in evaluating risk of mortality in hospitalization of patients with AMI. Furthermore, elevated serum sclerostin and OPG may be accompanied with a worse prognosis in patient with AMI. Therefore, bone-related proteins could exert a potential role in early risk stratification and prognosis assessment. However, the underlying mechanism between serum bone-related proteins and atherosclerosis remains uncertain, and further studies are needed to confirm our findings.

## Data Availability

The data that support the findings of this study are available from the corresponding author upon reasonable request.

## Abbreviations

AMI: Acute myocardial infarction
CAD: Coronary artery disease
OPG: Osteoprotegerin
ACS: Acute coronary syndrome
Wnt: Wingless-type mouse mammary virus-integration site
VSMCs: Vascular smooth muscle cells
eGFR: Estimated glomerular filtration rate
ECG: Electrocardiography
cTNI: Cardiac troponin I
PCI: Percutaneous coronary intervention
BMI: Body mass index
HDL-C: High density lipoprotein cholesterol
NT-proBNP: N terminal pro B type natriuretic peptide
GRACE: The Global Registry of Acute Coronary Event
MACE: Major adverse cardiovascular events
CABG: Coronary artery bypass grafting
ROC: Receiver operating characteristics
AUC: Area under the ROC curve
LDL-C: Low density lipoprotein cholesterol
FPG: Fasting plasma glucose
HbA1c: Hemoglobin A1c
TG: Triglyceride
UA: Uric acid
WBC: White blood cell
TC: Total cholesterol
HR: Hazard ratio
CI: Confidence intervals
LRP: Low-density lipoprotein receptor-related proteins
cTNT: Cardiac troponin T
